# Research Advance in Intestinal Mucosal Barrier and Pathogenesis of Crohn's Disease

**DOI:** 10.1155/2016/9686238

**Published:** 2016-08-29

**Authors:** Kuan Wang, Lu-yi Wu, Chuan-zi Dou, Xin Guan, Huan-gan Wu, Hui-rong Liu

**Affiliations:** ^1^Shanghai Institute of Acupuncture-Moxibustion and Meridian, Shanghai 200030, China; ^2^Shanghai Shuguang Hospital Affiliated with Shanghai University of Traditional Chinese Medicine, Shanghai 201203, China

## Abstract

To date, the etiology and pathogenesis of Crohn's disease (CD) have not been fully elucidated. It is widely accepted that genetic, immune, and environment factors are closely related to the development of CD. As an important defensive line for human body against the environment, intestinal mucosa is able to protect the homeostasis of gut bacteria and alleviate the intestinal inflammatory and immune response. It is evident that the dysfunction of intestinal mucosa barriers plays a crucial role in CD initiation and development. Yet researches are insufficient on intestinal mucosal barrier's action in the prevention of CD onset. This article summarizes the research advances about the correlations between the disorders of intestinal mucosal barriers and CD.

## 1. Introduction

CD and ulcerative colitis (UC) are inflammatory bowel disease (IBD). As a chronic, nonspecific, and granulomatous bowel disease, CD often occurs in the whole layer of intestinal wall, and, mostly, its lesions are segmentally and asymmetrically distributed. It may appear in any part of the gastrointestinal tract, especially in terminal ileum and adjacent colon [1]. CD has a long course as well as poor prognosis. Moreover, it occurs refractorily and repeatedly. According to the epidemiological investigation [2–7], the incidence of CD is higher in some developed countries in Europe and the United States and is increasing in Asia areas (especially in China). Nowadays, the etiology and pathogenesis of CD have not yet been fully recognized. Various genetic, immunologic, and environmental factors have been proved to be associated with the occurrence and development of CD, among which the immunologic factor is considered to be one of the most important factors [8–11]. The intestinal mucosal barrier dysfunction caused by immune abnormalities and infection is critical in the pathogenesis of CD. In this article, we mainly summarized the research advances about the correlations between the disorders of intestinal mucosal barriers and CD, including mechanical, chemical, immune, and biological barriers.

## 2. The Structure and Function of Intestinal Mucosal Barrier

Intestinal mucosal barrier is composed of mechanical barrier, chemical barrier, immune barrier and biological barrier, constituting a defensive barrier between the human body and the surrounding environment. The mechanical barrier mainly consists of intestinal epithelial cells and epithelial tight junctions. Tight junction (TJ) is the main connection form between intestinal mucosal epithelial cells, and it also plays an important role in maintaining the integrity of structure and normal function of intestinal mucosal barrier. Chemical barrier is made up of many chemicals such as digestive acid secreted by gastrointestinal, digestive enzymes, lysozyme, mucopolysaccharides, glycoproteins, and glycolipids. Therefore, it is involved in the process of bacteriolysis to inhibit the invasion of pathogenic bacteria. Gut-associated lymphoid tissue (GALT) and secretory immunoglobulin A (SIgA) as well as some special cells (such as macrophages, natural killer cells, and intraepithelial lymphocytes) constitute the immune barrier, which is an important guarantee for the intestinal immunity homeostasis via identifying the autoantigens and exogenous antigens to regulate the immune response. Actually, biological barrier is a mutually dependent and interrelated microecosystem. It is mainly composed of the resident intestinal flora, among which obligate anaerobe is the dominant bacterial community. Intestinal mucosal barrier is a barrier constituted between the organism and the surrounding environment. Those four barriers have distinguished structures and regulatory mechanism and each plays a different role in biological function. Intestinal mucosal barrier can effectively maintain the balance between pro- and anti-inflammatory factors and prevent pathogenic microorganism from entering into the tissues to keep the body healthy [12–15]. An important component of intestinal homeostasis and inflammation is the integrity of the intestinal barrier and the dysfunction of intestinal mucosal barrier is key to the occurrence of CD; therefore, maintaining the integrity of the intestinal mucosal barrier is of great significance in clinical CD prevention and treatment.

## 3. CD and Mechanical Barrier

The intestinal epithelial tight junction (TJ) is an important part of the intestinal mechanical barrier, and it is indeed the most essential structure to maintain the function of mechanical barrier. TJ is mainly composed of occludin, claudin, junction adhesion molecules (JAMs), and ZOs [16–18], among which claudin is the main frame protein, as the transmembrane protein in the claudin protein family, claudin-1, always plays a significant role in maintaining the integrity of intestinal epithelial TJ and the normal function of intestinal mechanical barrier [19, 20]. TJ possesses many protein complexes which are able to regulate the paracellular permeability. The intestine infection may be followed by TJ impairment, leading to intestinal epithelial permeability increase and intestinal mucosal barrier damage. This has been recognized as the key process to initiate the intestinal inflammation as well as the immune reaction. IFN-gamma can affect the expression of claudin-2 and occludin proteins through different mechanisms, like inducing the apoptosis of intestinal epithelial cells and destroying the integrity of intestinal epithelial TJ, eventually leading to IBD [21–23]. The aberrant increase of TNF-alpha level in the colonic mucosa of CD significantly reduced expression of occluding, claudin-1, and ZO-1 protein and mRNA and finally resulted in the structure impairment and TJ dysfunction. A new study [24] has also suggested that the inhibition of p38MAPK/p53 signaling pathway can increase the expression of TJ proteins (ZOs, protein-1, and occludin) and alleviate injury to the intestinal mucosal barrier.

## 4. CD and Chemical Barrier

The mucus secreted by gastrointestinal tract together with various other substances forms the intestinal mucosal chemical barrier, which is the key component of the body's natural immune system. Among all these substances, mucus is the most effective one in protecting the surface of intestinal mucosa. Intestinal mucous layer consisting of goblet cells and mucin (MUC) secreted by intestinal epithelial cells is the first defensive line to resist against extraneous pathogen through protecting and lubricating intestine. The intestinal mucous layer can be divided into external mucous layer which provided a suitable symbiotic environment for the gut microbiota and the internal mucous layer which protected the integrity of intestinal mucosal barrier by preventing microorganism from invading intestinal epithelium. Normally, only when the body is in a disease state caused by some abnormal factors could the bacteria penetrate the internal mucous layer and destroy the intestinal epithelium subsequently. MUC is not only the main component of the intestinal mucous layer but also the most important functional unit in mucus [25]. The mucin in the colorectum can be mainly divided into MUC1, MUC2, MUC3A, MUC3B, MUC4, MUC13, and MUC17, among which MUC2 is the most important one [26, 27]. It has been proven that the allelic polymorphism of MUC1 and MUC2 is closely associated with CD. Moreover, a large number of inflammatory cytokines (such as IL-4, IL-6, IL-13, TNF-alpha, and IFN-gamma) can promote the secretion of MUC in epithelial cells cultured* in vitro* [28, 29]. Studies [30, 31] have shown that MUC2 has direct antibacterial effect by forming the antiprotease substrates to defend the bacterial invasion. In Th1 and Th2 colitis rats model, MUC1 could regulate Th17 immune response and inhibit inflammatory response as Th17 cytokines stimulated MUC1 generation whose negative feedback regulated Th17 generation, so as to downregulate T17 mediated immune response, finally inhibiting the inflammatory reaction [32].

## 5. CD and Immune Barrier

The immunological factor has been considered to be the key factor in the occurrence and development of CD. Intestinal mucosal immune barrier is essential for maintaining intestinal immune homeostasis. GALT is made up of lymphoid nodule, free lymphoid tissue, plasma cells, and the intestine-related tissue composed of lymphocyte in the epithelium. GALT is an important immune organ to maintain the integrity of intestinal mucosal barrier. SIgA secreted immune globulin with diverse functions and is a main antibody that plays an important role in effects of anti-infection and immunomodulation in defense system of mucosa. A related study [33] found that the level of SIgA expression in patients with CD decreased obviously compared to the normal controls, and its level was negatively correlated with the severity of CD. It can be concluded that the intestinal mucosal immune system will lose the immune tolerance ability when the pathogenic bacteria and its antigen intrude into body; then the pathogen invades the intestinal epithelium and destroys the intestinal mucosal barrier. Paneth cells (PC), which are the typical cells of small intestine, are vitally important components of intestinal mucosal barrier and the main effector cells of small intestinal mucosal barrier. PC contain a variety of antibacterial material such as defensins, lysozyme, and SIgA [34–36], in which both defensins and lysozyme have the spectrum antimicrobial activity and can promote the innate immune response by killing the bacteria and keeping the steady state of intestinal flora [37]. Antibacterial peptide is alkaline peptide and maintains the balance of intestinal flora and the integrity of intestinal mucosal barrier via interacting with the bacteria in mucosal surface to keep endothelial cells away from being invaded [38–40]. Lysozyme can hydrolyze the peptidoglycan in pathogenic bacteria and change the osmotic pressure between intracellular and extracellular states. Recent researches [41, 42] indicate the therapeutic potential of lysozyme on various systemic inflammatory diseases. The functional lysozyme can also be used as a tracking reagent for microbial population in antibacterial tests. Besides, the nucleotide-binding oligomerization domain 2 (NOD2) expressed in PC could identify the bacterial peptidoglycan and kill the pathogens through the generation of antimicrobial peptide and induction of bacteria autophagy in the cell as well as the modulation of immunity [43, 44]. Researches [45, 46] have shown that the NOD2 gene mutation in CD may increase the susceptibility of the disease through influencing the interaction between ileal microbes and intestinal mucosal immunity. T cell immunoglobulin and mucin domain-3 (TIM-3), the newly discovered T cell immunoglobulin and mucin domain, is expressed specifically and merely on surface of the mature and active T cells. TIM-3 may be involved in the process of regulating T cells proliferation and activation and inhibiting the immune response mediated by Th1 cells [47–49]. TIM-3 plays an important role in chronic inflammatory and autoimmune diseases in humans [50, 51] and is a possible candidate for the treatment of disease in clinic. Simultaneously, TIM-3 also plays a critical role in regulating the activities of macrophages, dendritic cells, monocytes, natural killer cells, mast cells, and endothelial cells. The level of TIM-3 expression in Th1 cells of the intestinal mucosa in CD patients increased more obviously than in healthy persons, as decreasing the expression level of TIM-3 in Th1 cells may provide a new cure for a number of chronic inflammatory diseases in clinical practice [49]. Furthermore, regulating the levels of Th17 and Treg cells in intestinal mucosa could alleviate the intestinal inflammatory response and improve the integrity of intestinal epithelium mucosal barrier via increasing the expression of TJ proteins and mRNA and inhibiting the apoptosis of intestinal epithelial cells [52–55]. The severity of colitis is closely related to the level of IL-18 in intestinal epithelial cells, and, as a microbial modulator, the NOD-like receptor protein 6 (NLRP6) inflammasome can drive the microbial community stability [56–58]. Both IL-18 and NLRP6 inflammasome have key roles in maintaining homeostasis and intestinal barrier function.

## 6. CD and Biological Barrier

The biological barrier is constituted by normal flora and deposited in intestinal mucosa to maintain the integrity of the intestinal mucosal barrier. Normally, the microecological environment in intestine maintains homeostasis through the interdependence and mutual restrictions between probiotics and pathogenic bacteria. IBD is accompanied with alteration of intestinal flora, which could induce intestinal infection when body is affected by abnormal factors [59–61]. Both the* Bifidobacterium* and* Lactobacillus* are the probiotics. On one hand, they could restrict the pathogenic bacteria; on the other hand, they could repair the damaged mucosal barrier by adjusting the level of inflammatory cytokines. A study had demonstrated that lactic acid bacteria could decrease the levels of IL-6, TNF-alpha, toll-like receptor 4 (TLR4), and NF- kappaB mRNA and increase the level of IL-10 mRNA observably at the same time [62]. In the feces of patients with CD, the amount of* bacteroid*,* Bacillus,* and* Streptococcus* were increased, while the amount of* Bifidobacterium* was decreased [63]. Prebiotics would protect the integrity of intestinal epithelium barrier by promoting the expression of ZO-1 and occludin protein [64, 65]. Studies [66–69] have shown that the normal gut microbiota could prevent bacteria from contacting with the intestinal epithelium, and probiotics could balance the intestinal flora in experimental colitis model of rats through regulating the intestinal mucosal barrier and the levels of related immune cells. Therefore, probiotics may repair the damaged mucosa and maintain the integrity of intestinal mucosal barrier.

## 7. Conclusion

The mechanical, chemical, immune, and biological barriers play important role in protecting the gut against bacteria homeostasis, regulating the intestinal immune response and reducing the inflammatory response. Yet the comprehensive and systematic researches are insufficient on intestinal mucosal barrier's action in the prevention of CD onset. Therefore, it is of great significance to conduct more thorough studies and randomized controlled trials with largescale, multicentre, and high-quality. In addition, interventions by which to maintain the structural integrity and proper function of intestinal mucosal barrier are expected to be a rational and reliable approach in the prevention of CD in the future.
